# Systematic Investigation of Co-Crystallization Properties in Binary and Ternary Mixtures of Triacylglycerols Containing Palmitic and Oleic Acids in Relation with Palm Oil Dry Fractionation

**DOI:** 10.3390/foods9121891

**Published:** 2020-12-18

**Authors:** Veronique Gibon, Sabine Danthine

**Affiliations:** 1 Desmet Ballestra Group, R&D Center, Belgicastraat, 3 Bus 1-2, B-1930 Zaventem, Belgium; 2 Science des Aliments et Formulation, Gembloux Agro-Bio Tech, Liège University, 5030 Gembloux, Belgium; sabine.danthine@uliege.be

**Keywords:** palm oil, dry fractionation, binary phase diagrams, ternary phase diagrams, tripalmitin, 2-oleo-dipamitin, 1-oleo dipalmitin, 1-palmito-diolein, molecular interactions

## Abstract

This work investigates the molecular interactions within the main triacylglycerols constitutive of palm oil, all having a key role in the multi-step dry fractionation process. Identification of these interactions is possible thanks to the establishment of binary and ternary phase diagrams, using differential scanning calorimetry (DSC) and powder X-ray diffraction (XRD) at variable temperature. The following systems were selected: PPP-POP, PPP-OPP, PPP-POO, POP-OPP, POP-POO, OPP-POO, PPP-POP-POO and PPP-OPP-POO (P: palmitic acid and O: oleic acid), and analyzed in direct mode (heating at 5 °C/min., after melting and quenching at −60 °C), and after tempering for three months at 20 °C (tempered mode). DSC makes it possible to bring out crystallization and melting phenomena associated to polymorphic transitions, which are further characterized (crystalline forms) by XRD. The results show that unsaturated are poorly soluble in fully saturated triacylglycerols, that the intersolubility decreases in proportion to the number of unsaturated fatty acids, that positional isomerism (POP/OPP) has a major impact, that OPP may induce formation of molecular compounds and that co-crystallization properties are highly modified by tempering depending on the polymorphic properties of the systems. This provides a better understanding and allows for effective control of the palm oil dry fractionation process.

## 1. Introduction

Palm oil is the most widely consumed edible oil, with Indonesia and Malaysia by far the largest producing countries. Most of the oil is used for food applications such as frying, cooking, salad oils, margarines, spreads, confectionary fats, etc. Palm oil is a semi-solid fat with a melting point above 35 °C. Both palmitic (P: C16:0) and oleic (O: C18:1) fatty acids contribute for about 85% of the total fatty acid composition. Triacylglycerols (TAGs) can be classified according to their saturation: tri-saturated (StStSt), mono-unsaturated (St_2_U), di-unsaturated (StU_2_) and tri-unsaturated (UUU). In palm oil, tripalmitin (PPP), oleo-dipalmitin (P_2_O), palmito-diolein (PO_2_) and trilolein (OOO) are the most abundant TAGs of each class. Mono- and di-unsaturated TAGs are found to be symmetrical (StUSt and UStU) or asymmetrical (UStSt and StUU); their relative proportion in crude palm oil is generally constant [1,2]. However, the StUSt/UStSt ratio can be altered during palm oil refining if the deodorization takes place at temperatures that are too high or for a too long time: this leads to an increase of OPP at the expense of POP by sn-2 acyl migration [3]. Random interesterification (chemical or enzymatic) and enzymatic remediation of palm oil also induce migration of palmitic acid in sn-2 position and modify the POP/OPP ratio [4,5,6]. Palm oil has a remarkable crystallization potential, making it a prime candidate for dry fractionation which is an industrial modification process allowing extension of its use in food formulation. Multi-step dry fractionation of refined palm oil creates a wide variety of solid and liquid fractions for food applications. Moreover, palm oil and its fractions are now widely used to meet the trans-free fat requirements of the food industry [7,8,9,10,11]. These fractions are particularly enriched or depleted in StStSt, St_2_U, StU_2_ or UUU; their TAG composition can be quantified by reverse phase high performance liquid chromatography (RP-HPLC). The iodine value (IV) refers to the unsaturation degree of a fat and can be determined by titration or calculated from gas chromatography (GC); typical IV for palm oil ranges from 51 to 53 while the IV of the highest melting point fraction (super-stearin) is usually below 15 and the IV of the most liquid fraction (top-olein) can exceed 72 [12,13]. Melting and crystallization properties of palm oil and fractions can be determined by analytical techniques like pulsed nuclear magnetic resonance (p-NMR) or differential scanning calorimetry (DSC) [14,15]. The molecular interactions between the different triacylglycerols in palm oil can be studied using DSC combined with powder X-ray diffraction (XRD), by establishing phase behavior diagrams. Indeed, in the context of lipids, phase behavior describes the transitions of a fat from liquid to solid state (and the reverse), the polymorphism of the solid phase, and the transition between solid phase polymorphic forms. Describing fat phase behavior is of significant interest across numerous application areas [16,17]. One of the best approaches to elucidate the behavior of complex triacylglycerol systems is to simplify the matrix and to emphasize the main components, in the case of palm oil: PPP, POP, OPP and POO.

Some phase diagrams are already reported in the literature as recently reviewed by Machridachis-Gonzales et al. [18]. Unfortunately, a deep comparative study of binary and ternary systems including the main TAGs of palm oil (PPP, POP, OPP and POO) all obtained under similar and particular conditions, in direct and in tempered modes, is however missing. The data available in the literature relating to systems containing palmitic and oleic acids are summarized below. Gibon et al. compared PPP/PSP and PPP/POP binary systems, focusing on the influence of fatty acid unsaturation (stearic vs. oleic) [19,20,21]. Minato et al. reported some structural aspects with the use of synchrotron X-ray radiation [22]; they claimed that the PPP/POP system shows immiscible monotectic properties in both metastable and stable forms, while Gibon et al. reported that the metastable forms were partly miscible. For the same system, Lu et al. reported the formation of solid solution and eutectic interaction [23]. The observed differences are obviously linked to the different experimental conditions (namely fast vs. low cooling/heating rates). Minato et al. described the POP/OPP binary system and its phase behavior in metastable and in the most stable forms obtained after 90 days tempering at 26 °C [24]. The formation of a molecular compound [25] indicating specific interactions through the acyl chains for a 50/50 concentration ratio was reported. This induced, on each side, two juxtaposed monotectic behaviors (OPP/molecular compound and molecular compound/POP). These authors proposed a model structure for this molecular compound, involving the separation of palmitoyl-chain leaflet and palmitoyl-oleoyl mixed-acid-chain leaflet in the double chain length structure. In 1963, Moran already suggested the formation of a molecular compound for the mixture POP/OPO and estimated its occurrence at 50/50 concentration ratio [26]. Later, this system was further investigated by Minato et al.; these authors also described a molecular compound at the 50/50 concentration ratio, with two juxtaposed monotectic behaviors (POP/molecular compound and molecular compound/OPO) [27]. This was further confirmed by Ikeda et al., who investigated the occurrence of this molecular compound under diluted conditions [28]. Miura and Konishi investigated the effect of temperature cycling (5–20 °C) on granular crystal formation in a model margarine; in this context, they studied the POP/POO binary blends: they pointed out that when POP and POO were mixed, the β polymorph did not emerge [29]. Some years later, Zhang et al. reported that the tempered POP/POO binary mixture exhibits immiscible eutectic behavior without molecular compounds in the tempered system, contrarily to POP/OPP and POP/OPO binary systems [30]. The same eutectic behavior (POP/POO) was also reported by Lu et al., as well as for the PPP/POO system, when using slow cooling and heating rates [23]. Mizobe et al. investigated the mixing behavior of the enantiomers S-OPP and R-PPO during cooling and heating. This system has a eutectic interaction due to the different polymorphic structures of the two enantiomers [31]. Recently, Bayes-Garcia et al. examined three binary mixtures: POO-OPO, OPP-POO and OPP-OPO; their research work focused on molecular compound formation and stability [25]. The importance of the stereochemistry of asymmetrical triacylglycerols for understanding their mixing behavior was again revealed. Triacylglycerol symmetry is an important factor that is to be considered when it comes to solid-state miscibility.

The approach of the work reported here is rather different; indeed it focuses on a global comparison of the crystallization behaviors of PPP-POP, PPP-OPP, PPP-POO, POP-OPP, POP-POO, OPP-POO, PPP-POP-POO and PPP-OPP-POO blends. It systematically explores and compares these phase diagrams obtained in the same thermal conditions, thanks to DSC combined with variable temperature powder XRD. Results of the study allow clarification of a series of phenomena observed during the crystallization of palm oil in dry fractionation.

This differs from all the fundamental investigations previously reported in the literature by its direct link with an industrial process, in this case the dry fractionation of palm oil; such an approach had not been reported yet.

## 2. Experimental Conditions

### 2.1. Material

Tripalmitin (PPP) was a Supelco (Bellonfonte, PA, US) product. Purity, verified by gas chromatography (GC), was above 99%. Furthermore, 2-oleo-dipalmitin (POP), 1-oleo-dipalmitin (OPP) and 1-palmito-diolein (POO) were synthesized and purified at Gembloux Agro-Bio Tech (Université de Liège, Gembloux, Belgium) based on a procedure described by Elisabettini et al. [32]; purity, verified by GC, was above 99%. Standards for HPLC were from Larodan (Solna, Sweden) (OOO, POO, POP) and Sigma (St Louis, MO, USA) (PPP) (purity above 99%).

### 2.2. Reverse Phase High Performance Liquid Chromatography (RP-HPLC)

The triacylglycerol composition was analyzed by reverse phase HLPC based on the AOCS Ce 5b-89 recommended method which does not distinguish between positional isomers [33]. Analyses were performed with a Waters HPLC system, equipped with two stainless steel Nova-Pak C18 columns (4 μm, 3.9 × 150 mm). The mobile phase was an isocratic solvent mixture of acetone and acetonitrile (62.5/37.5 *v/v*) with a flow rate of 1.2 mL/min; the injection volume was 20 µL. The samples were dissolved in methanol/chloroform (1/1 *v/v*) and a differential refractometer was used for the detection. This method allows quantification of individual triacylglycerols which are eluted according to their partition number (PN). PN = CN − 2(DB), where CN is the total carbon number and DB is the total double bonds for each fatty acid. For example, in the group of PN = 48, OOO, PO_2_, P_2_O and PPP elute separately and in sequence. The assignment was confirmed by comparing retention times with those of some standards (PPP, POP, OPP, OOO). Peaks were integrated with Empower Pro “Apex Track” algorithm; peak areas below 4000 area counts (equivalent to approximately 0.04% of the total peak area) were not taken into account.

### 2.3. Iodine Value (IV) 

The iodine value was determined using gas chromatography (GC) based on the AOCS Ce-1h-05 recommended method [34]. Analyses were performed with an Agilent 6890N GC equipped with an on-column injection system. The column was BPX 70 (70% cyanopropyl polysilphenylene-siloxane, 0.25 mm/60 m, film thickness 0.25 mm) from SGE. The flame-ionization detector (FID) was set at 280 °C. The carrier gas was H_2_ at a pressure of 9.08 PSI and a flow of 0.7 mL/min. The injection volume was 1 µL. Before injection, the triacylglycerols were converted into fatty acid methyl esters based on the AOCS Ce 2-66 recommended method and the iodine value was calculated according to the AOCS Cd 1c-85 recommended method [35,36].

### 2.4. Differential Scanning Calorimetry (DSC)

DSC measurements were performed with a Setaram DSC 111 (Setaram, Lyon, France), using liquid nitrogen as cooling device. Samples of about 10 mg were used, with an empty pan as reference. Calibration was made with indium and nitrogen was used as purge gas. Melting profiles were recorded at 5 °C/min according to two different procedures (direct mode and tempered mode).

Endothermic and exothermic temperatures reported in the phase diagrams correspond to maximal temperature of each peak (Tp).

#### 2.4.1. Direct Mode

Samples were first heated up to 80 °C for 10 min to ensure complete melting and thermal memory erasure. Afterwards, they were quickly frozen (quenched) at −40 °C (−25 °C/min) and kept for 10 min at this temperature for complete solidification. The reported melting profiles were recorded from −40 to 80 °C at a heating rate of 5 °C/min.

#### 2.4.2. Tempered Mode

The DSC profiles of the same samples were recorded after three months of tempering at 20 °C. The objective of this tempering was not to stabilize all blends in their most stable polymorphic form but to compare crystallization properties evolution in identical operating conditions. For that purpose, samples were first heated up to 80 °C for 10 min, quickly frozen (quenched) at −40 °C (−25 °C/min), kept for 10 min at this temperature and heated to 20 °C at 5 °C/min. DSC pans were then removed and tempered in an oven stabilized at 20 ± 1 °C. After three months, the pans were further placed in the DSC, quickly frozen (quenching) at −40 °C (−25 °C/min), kept for 10 min at this temperature and melted; the reported melting profiles were recorded from −40 to 80 °C at a heating rate of 5 °C/min.

### 2.5. Variable Temperature Powder X-ray Diffraction (XRD)

Polymorphism was investigated by powder X-ray diffraction. A Philips (Eindhoven, The Netherlands) PW 1050 diffractometer (K_α Cu_: 1.54178 Å) equipped with a temperature control system (Philips PM 2522 A digital VAΩ meter and Pt probe) was used. Temperature control was performed with a TTK Anton Paar system (Anton Paar, Graz, Austria) coupled with cold ethanol circulation using a Huber HS 60 cryostat (Huber, Milano, Italy). Aluminum sample pans contained about 100 mg of material. A gaseous nitrogen flow prevented condensation of water during measurements at low temperatures. Polymorphism was identified at selected temperatures based on the corresponding DSC profiles (direct and tempered modes). Short (15 to 27°θ; 0.02°2θ/s) and long (1 to 15°θ; 0.02°2θ/s) spacings were analyzed in separate runs. Short spacings make it possible to differentiate polymorphic forms (sub-α, α, β^′^, β…) while long spacings give information on the type of stacking (double-chain length L-2 or triple-chain length L-3) [37,38]. This nomenclature is largely used for linear molecules like TAGs in their different polymorphs. The long spacing value is linked to the way the molecules stack up and is associated with the carbon number and the tilting of the molecule [39]. The L-2 structures are usually characterized by values comprised of between 30 and 52 Å [40,41]. A schematic representation of the different kind of structure in crystallized fats is presented in Campos et al. [42].

### 2.6. Phase Diagrams

The investigated binary and ternary mixtures were prepared by mixing melted pure triacylglycerols in the appropriate weight, directly in the DSC pans; pans were immediately sealed. The compositions reported are expressed as weight fractions. The phase diagrams were constructed thanks to DSC heating thermograms (melting and transition temperatures) and X-ray powder diffraction patterns (short and long spacings) The black circles in the binary diagrams correspond to melting temperatures (endotherms) and the open squares on circles to transition temperatures (exotherms). The ternary diagrams are presented as melting iso-lines; they connect high melting and low melting points of binary and ternary systems.

## 3. Results

### 3.1. Triacylglycerol Composition of Palm Oil and Fractions

**Figure 1:**
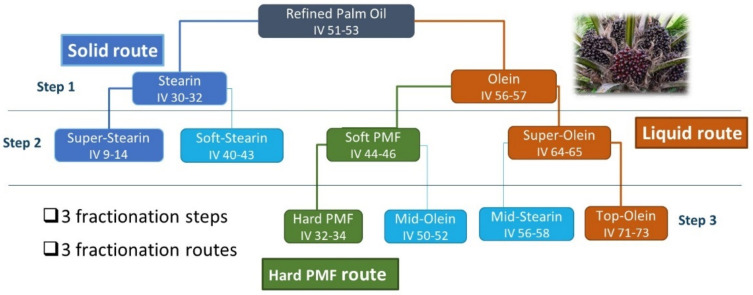
Multi-step dry fractionation of palm oil (PMF: palm mid fraction).

The industrial multi-step dry fractionation process of palm oil involves three steps and can be conducted along three routes (Figure 1): the liquid route, the solid route and the hard palm mid fraction (PMF) route. All fractions have specific IV and TAG composition. PPP, P_2_O and PO_2_, the main representatives of mono-saturated, mono- unsaturated and di-unsaturated TAGs, are reported in Table 1 for palm oil and some fractions. The liquid fractions (olein, super-olein and top-olein) are gradually depleted in P_2_O; the solid fractions (stearin and super-stearin) are particularly enriched in PPP and the palm mid fractions concentrate P_2_O.

In practice, PPP and P_2_O play an essential role in what concerns the physicochemical characteristics of the palm liquid fractions: improving the cold stability is possible thanks to PPP removal in the olein and to P_2_O reduction in the super- and top-olein [13]. On the other side, a minimal amount of PPP in the olein is necessary to ensure a stable crystallization allowing a reproducible fractionation process for super-olein production [43]; this is possible by adding a few percent of palm oil (PPP source) to the olein before starting the crystallization. Furthermore, there is a critical crystallization zone which goes along with the crystallization of P_2_O and its reduction in the super-olein; this is characterized by quite important heat release that cannot be easily balanced by the cooling medium of the fractionation equipment. A similar critical zone is observed during hard PMF production, sometimes limiting the performance of this route [44]. The POP/OPP ratio in the refined palm oil seems to play a predominant role in these phenomena, but it has not been fully elucidated yet. The analysis of the specific molecular interactions occurring in blends of pure triacylglycerols as described in the next part is useful to explain these observations.

### 3.2. Polymorphic and Melting Properties of Pure Selected Triacylglycerols: PPP, POP, OPP and POO

**Figure 2:**
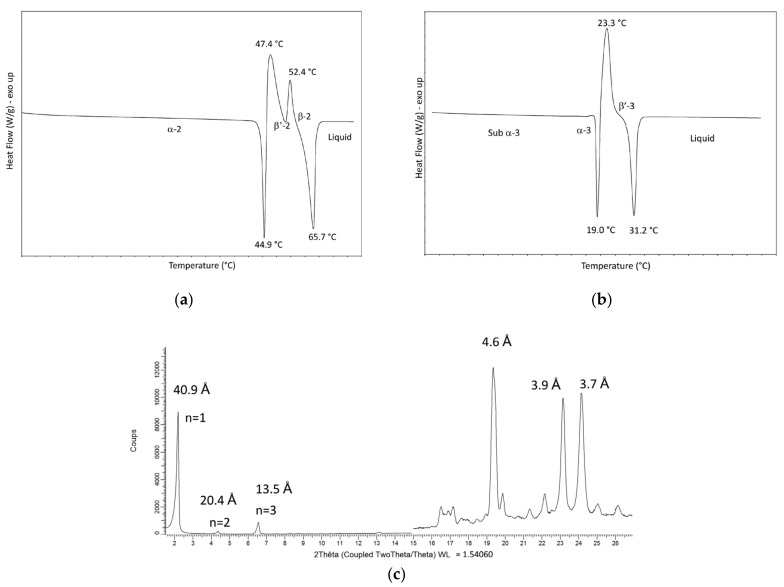
(**a**) Differential scanning calorimetry (DSC) melting profile of PPP in direct mode; (**b**) DSC melting profile of OPP in direct mode and (**c**) powder X-ray pattern of PPP in β-2 (n = order of diffraction).

Melting and transition temperatures and polymorphic forms observed in direct mode, last melting temperatures and polymorphic forms observed in tempered mode for pure PPP, POP, OPP and POO are presented in Table 2. As illustration, long and short spacings of POP, OPP and POO in direct mode are presented in Table 3; DSC melting profiles of PPP and OPP and X-ray powder diffraction pattern of PPP in β-2 are shown as example in Figure 2.

#### 3.2.1. Direct Mode

Upon heating, PPP shows a succession of endo- and exotherms corresponding to α-2 to β^′^-2 to β-2 transitions; the α-2 to β^′^-2 transition is melt-mediated, in accordance with literature data [45]. Only one β-2 form is observed in the used conditions although the existence of two slightly different β-2 forms has been reported by some authors [46]. In the same conditions, POP crystallizes in α-2 which transforms into β^′^-2; the α-2 to β^′^-2 transition does not appear to be melt-mediated. The X-ray powder diffraction pattern shows the existence of two types of β^′^ forms, both in double-chain length: sub-β^′^-2 and β^′^-2. These β^′^ forms were already reported by Lutton et al. [47], and later confirmed by Sato et al. [48]. The asymmetrical OPP crystallizes in L-3 stacking; three forms are observed: sub-α-3, α-3 and β^′^-3, in accordance with data reported by Minato et al. [24] and Mizobe et al. [31]. The α-3 to β^′^-3 transition is melt-mediated. POO crystallizes in sub-α-3, α-3 and β^′^-3; only one β^′^-3 is observed under the investigated conditions, contrary to Bayes-Garcia et al. who reported 2 distinct β^′^ forms [25,49]. The sub-α-3 to α-3 transition does not appear to be melt-mediated.

**Figure 3:**
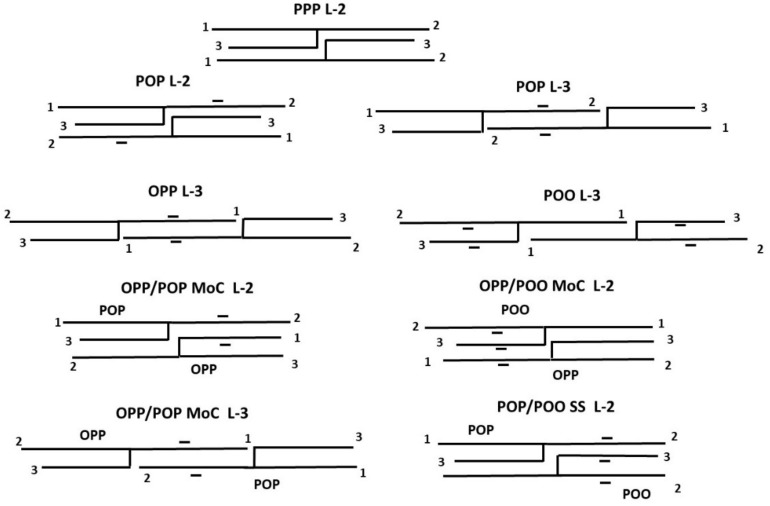
Proposed molecular arrangements for PPP L-2, POP L-2, POP L-3, OPP L-3, POO L-3, OPP/POP MoC L-2, OPP/POO MoC L-2, OPP/POP MoC L-3 and POP/POO SS L-2.

In direct mode, β polymorphism (double-chain length L-2) is only observed for the fully saturated PPP; the symmetrical POP crystallizes in double-chain length while the asymmetrical OPP and POO solidify in triple-chain length L-3. Various molecular arrangements derived from powder XRD are proposed in Figure 3. Considering that the double-chain length structures of POP, oleic and palmitic fatty acids are neighbors; this tends to transform with time into triple-chain length where the oleic and palmitic chains are in separate areas. The triple-chain length structures of OPP and POO are different; for OPP, the oleic chains pile up in the middle, while for POO, the palmitic chains meet in the middle (Figure 3). For this reason, the oleic chains of OPP tend to be more mobile which contribute to significantly enlarged long spacings values compared to the ones of POO (Table 3).

#### 3.2.2. Tempered Mode

During tempering, PPP remains in α form; therefore, the polymorphic transitions observed in tempered mode are α-2 to β^′^-2 to β-2; the last melting point in β-2 is almost not changed. The final melting temperatures evolve from 27.5 °C for POP in β^′^-2 and 31.2 °C for OPP in β^′^-3, to 34.2 °C for POP in β-3 (Δ = 6.7 °C) and to 32.6 °C for OPP in a more compact β^′^3 form (Δ = 1.4 °C) (Figure 3).The β-3 form of POP was already reported by Sato et al. [36], who also highlighted a γ metastable form which was not observed with the used conditions. As the tempering temperature (20 °C) is above the last melting temperature of POO, no modifications in the polymorphic forms and melting profile are observed in tempered mode.

### 3.3. Binary Phase Diagrams

In order to elucidate the co-crystallization properties of PPP, POP, OPP and POO, the next binary systems: PPP/POP, PPP/OPP, PPP/POO, POP/OPP, POP/POO and OPP/POO were studied both in direct and in tempered mode. The fatty acids involved are differentiated by the chain length (C:16 vs. C:18) and the presence of a cis double bond (O vs. P), the positional isomerism and the number of mono-unsaturated fatty acids on the glycerol.

#### 3.3.1. Direct Mode

**Figure 4:**
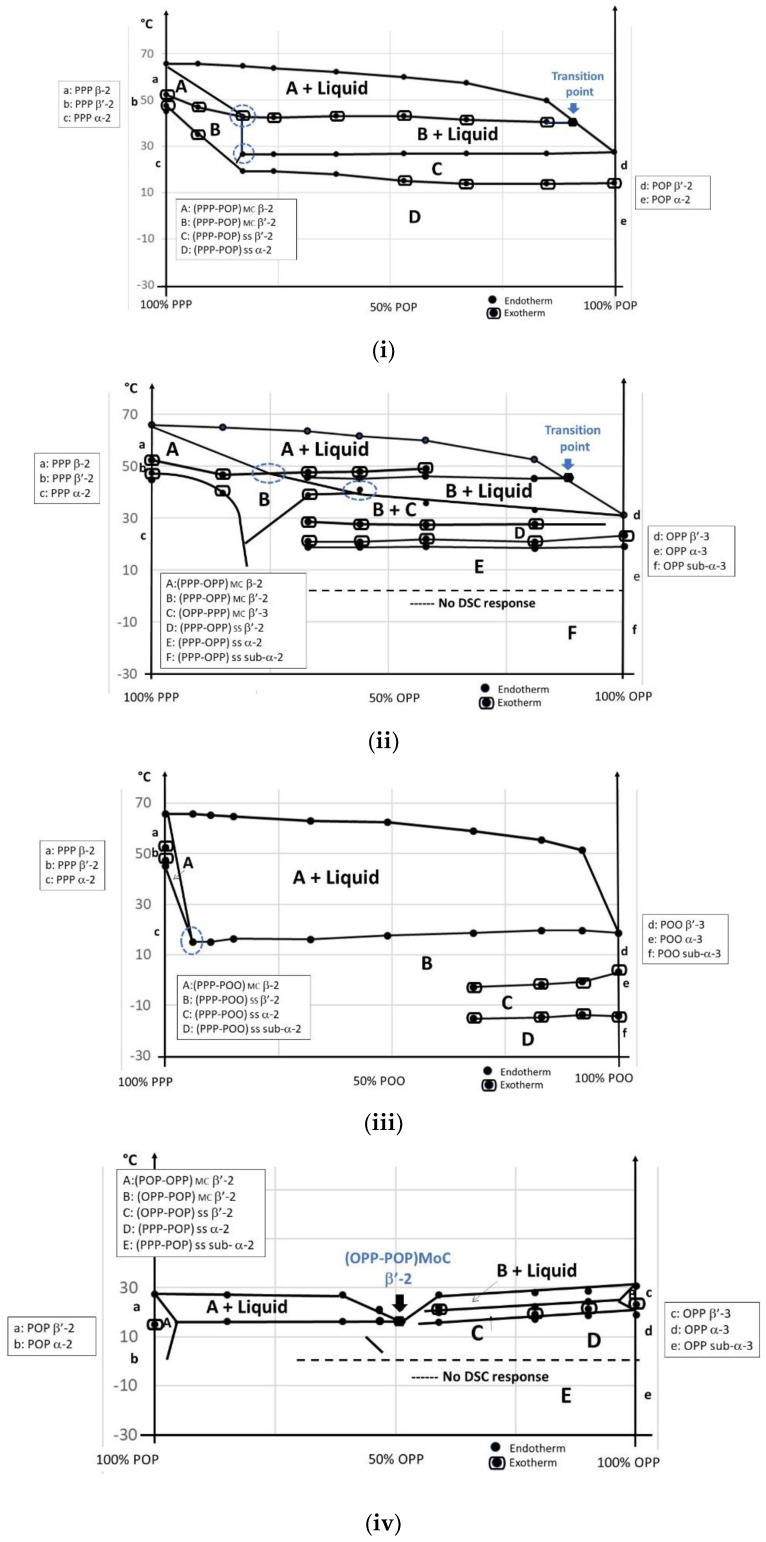

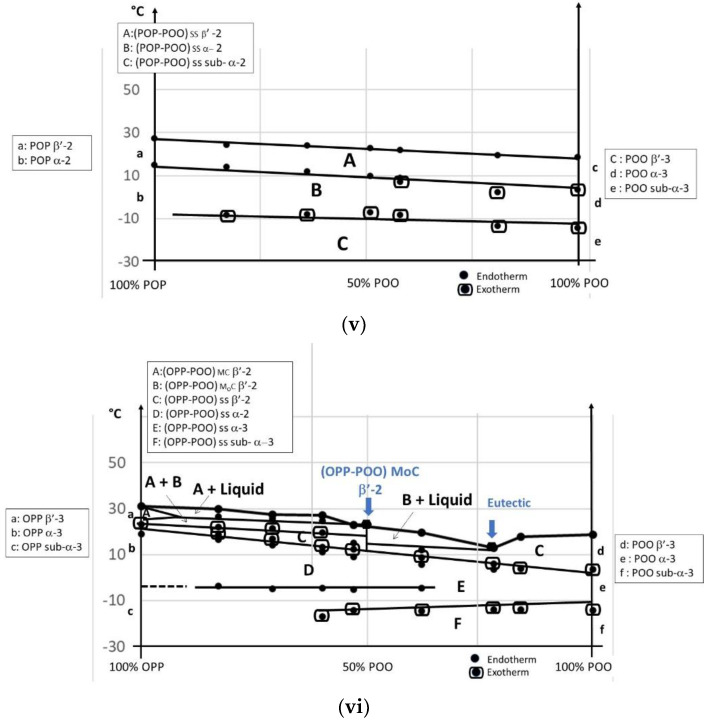
(**i**) (PPP-POP) phase diagram; (**ii**) (PPP-OPP) phase diagram; (**iii**) (PPP-POO) phase diagram; (**iv**) (POP-OPP) phase diagram; (**v**) (POP-POO) phase diagram and (**vi**) (OPP-POO) phase diagram, in direct mode. SS: solid solution; MC: mixed crystal; MoC: molecular compound.

The PPP-POP, PPP-OPP, PPP-POO, POP-OPP, POP-POO and OPP-POO phase diagrams obtained in direct mode are presented in Figure 4.

##### PPP/POP

As pure components, these two triacylglycerols crystallize in double-chain length and the difference in melting point is higher than 35 °C. It is therefore not surprising that all the blends also solidify in L-2, showing monotectic behavior for the final melting. After quenching, PPP/POP blends solidify in α-2 as a solid solution (SS): (PPP-POP) _SS_ (D), which is transformed into β^′^-2 (C) upon heating. A monotectic line is observed at ~27 °C which corresponds to the melting of significant amounts of POP β^′^-2 and the occurrence of mixed crystals (MC): (PPP-POP) _MC_ in β^′^-2 (B) with liquid. These mixed crystals are then transformed into (PPP-POP) _MC_ β-2 (A) (transition point) before final melting. Quantities of POP co-crystallizing with PPP in (PPP-POP) _MC_ β^′^-2 and in (PPP-POP) _MC_ β-2 are ~15% (blue dotted circles).

##### PPP/OPP

The PPP/OPP phase diagram is somewhat similar to PPP/POP in that they are both of monotectic type (similar difference in melting point) with a β^′^-2 to β-2 transition of mixed crystals (transition point); however, contrary to POP, OPP exhibits an L-3 polymorphism. Despite this, after quenching, PPP/OPP blends first solidify in sub-α-2 as (PPP-OPP) _SS_ (F) which transforms into α-2 (E) and then β^′^2 (D), upon heating. In this case, exothermic DSC peaks are observed just below the monotectic line and attributed to a transformation of (PPP-OPP) _SS_ β^′^2 into a mix of (PPP-OPP) _MC_ β^′^-2 (B) and (OPP- PPP) _MC_ β^′^-3 (C), already in the solid state. This melts upon heating and (PPP-OPP) _MC_ β^′^-2 (B) further transforms into β–2 (A) before final melting. Quantities of OPP co-crystallizing with PPP in (PPP-OPP) _MC_ β^′^-2 and β-2 reach ~25–45% (blue dotted circles) that is far above the maximal content of POP in PPP.

##### PPP/POO

POO solidifies in L-3 like OPP but the difference in melting point with PPP is higher (superior to 45 °C); this makes the interactions also of monotectic type but the β^′^-2 into β-2 transition is no longer observed in the monotectic zone. The monotectic line (at ~18 °C) goes along with the occurrence of (PPP-POO) _MC_ (A) directly crystallizing in β-2. Indeed, after melting and quenching, PPP/POO blends solidify as (PPP-POO) _SS_ sub-α-2 (D) which transform into α-2 (C) and into β^′^-2 (B) upon heating; this solid solution quickly transforms into (PPP-POO) _MC_ β-2. Quantity of POO co-crystallizing with PPP in (PPP-POO) _MC_ β-2 is very low: ~5% (blue dotted circles).

##### POP/OPP

The difference in melting point between POP and OPP is lower than 4 °C. POP solidifies in L-2 while OPP does in L-3. Specificity of this binary phase diagram is a minimum observed in melting temperature at ~50/50 concentration ratio, which can be attributed to the formation of a molecular compound (OPP-POP) _MoC_ crystallizing in β^′^ double-chain length (melting point ~22 °C) (Figure 3). On the left side of this molecular compound (POP side), only two endotherms are observed; on the right side (OPP side), the blends show three endotherms and one exotherm. After melting and quenching, all blends crystallize in a sub-α-2 (E) which transforms into α-2 (D) upon heating. On the left side of the molecular compound, a clear melting line is observed at ~15 °C, which corresponds with the appearance of (POP/OPP) _MC_ in β^′^-2 (A) and liquid. On the right side of the molecular compound, the solid solution α-2 (D) transforms into (OPP/POP) _MC_ β^′^-2 (C) becoming β–2 later (B) in the presence of liquid.

##### POP/POO

Difference in melting point between POP and POO is ~15 °C; POP exhibit L-2 and POO L-3 polymorphism. Surprisingly, after quenching, the blends crystallize in L-2 as solid solution (Figure 3) all over the phase diagram and the binary system looks mostly simple when joining all the corresponding endotherms and exotherms; a complete solubility seems to occur in all the polymorphic forms: sub-α-2, α-2 and β^′^-2 (A, B, and C).

##### OPP/POO

This binary phase diagram is significantly more complex compared to POP/POO. The melting point difference is ~12 °C and both pure OPP and POO solidify in L-3. The OPP/POO binary system shows some peculiar crystallization features, and in this case, at two specific compositions: at 50/50 and 25/75 concentration ratios. The first peculiar behavior appears as a break in the melting curve (50/50) and the second (25/75) as a minimum. Melting behaviors on the left and right sides of the 50/50 concentration which correspond to a molecular compound (OPP/POO) _MoC_ β^′^-2 (Figure 3) are completely different. Similarities are observed between the left side of this 50/50 concentration (enriched in OPP) and the right side of the POP/OPP system (also enriched in OPP). At the left side, a line can be drawn at ~25 °C corresponding to the melting of (OPP/POO) _MoC_ β^′^-2 (B) and to the occurrence of (OPP/POO) _MC_ β^′^-2 (A) and liquid. Contrariwise, the right side presents a kind of eutectic interaction, another stable structure, at higher amounts of POO. A line can be drawn at ~12 °C corresponding to the melting of (OPP-POO) _SS_ β^′^-2 (C) and its transformation into (OPP/POO) _MoC_ β^′^-2 (B) and liquid. It should be noted that, although both triacylglycerols solidify in L-3 as pure, the triple chain length structure is only observed after quenching at low temperature, in sub–α and in α: (OPP-POO) _SS_ sub-α-3 (F) and (OPP-POO) _SS_ α-3 (E); this solid solution transforms into α-2 (D) and β^′^-2 (C) upon heating.

#### 3.3.2. Tempered Mode

**Figure 5:**
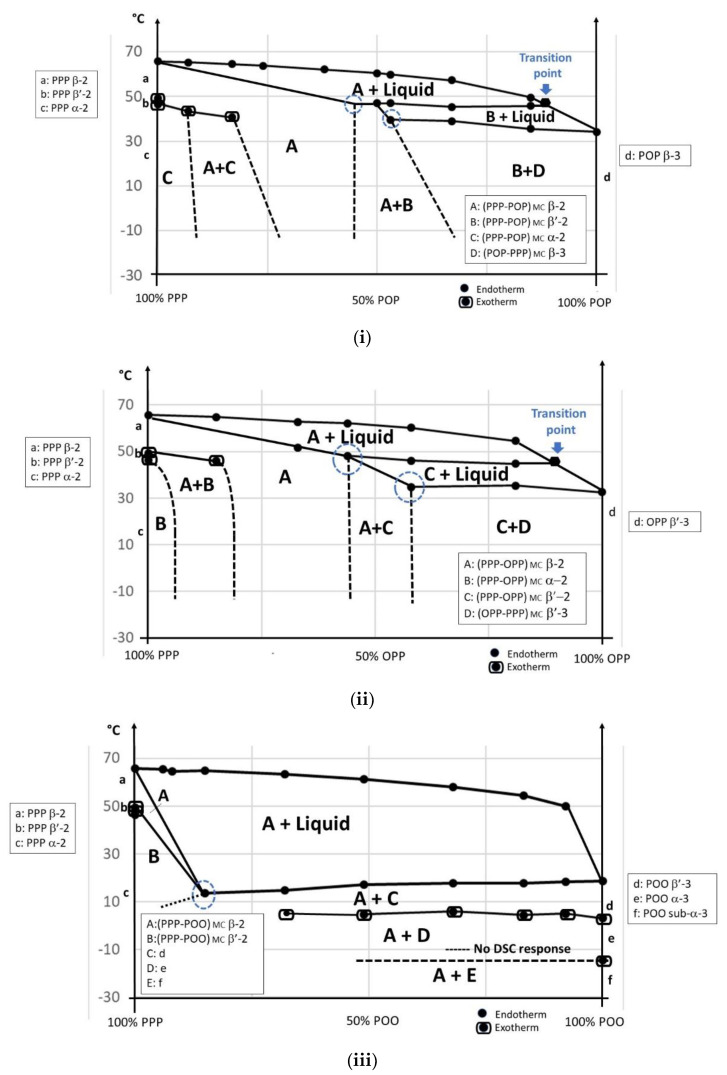

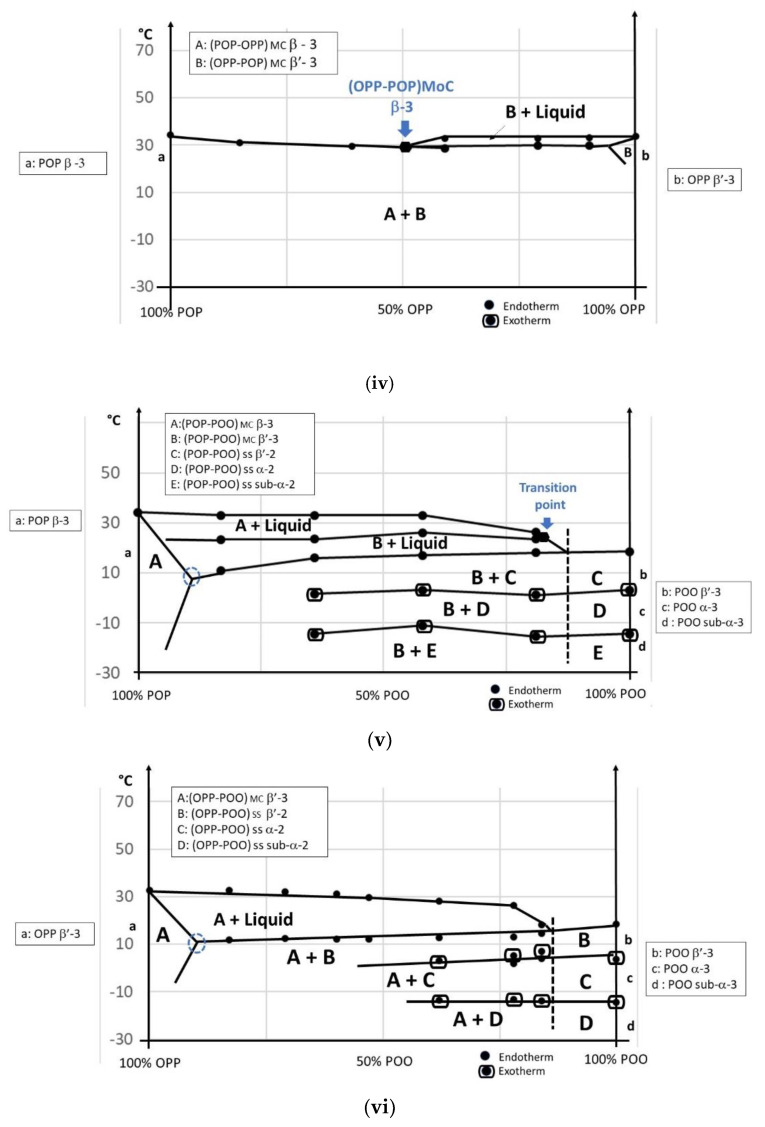
(**i**) (PPP-POP) phase diagram; (**ii**) (PPP-OPP) phase diagram; (**iii**) (PPP-POO) phase diagram; (**iv**) (POP-OPP) phase diagram; (**v**) (POP-POO) phase diagram and (**vi**) (OPP-POO) phase diagram, in tempered mode. SS: solid solution; MC: mixed crystal; MoC: molecular compound.

The PPP-POP, PPP-OPP, PPP-POO, POP-OPP, POP-POO and OPP-POO phase diagrams obtained in tempered mode are presented in Figure 5.

##### PPP/POP

With the tempered conditions used, the PPP/POP binary system remains of the monotectic type with a transition line at ~35 °C (and a transition point). POP transforms from β^′^-2 into β–3 while PPP stays in α-2 at the tempering temperature (20 °C). Global effect is that α-2 mixed crystals, β–2 mixed crystals and blends of α-2/β-2 mixed crystals and β^′^-2/β-2 mixed crystals are observed within well-defined areas (A, B, C and D). At high concentration (right side of the phase diagram), POP is excluded from (PPP-POP) _MC_ β^′^-2 and crystallizes separately as (POP-PPP) _MC_ β-3. Solubility of POP within (PPP-POP) _MC_ is comparable to direct mode: ~45–55% in the β^′^ and β forms (blue dotted circles).

##### PPP/OPP

The PPP/OPP binary system is also of monotectic type in the tempered mode, with a transition line at ~35 °C (and a transition point). The tempering used does not highly affect OPP polymorphism which remains in β^′^-3 form. As for PPP/POP binary system, α-2 and β-2 mixed crystals, blends of α-2/β-2 and of β^′^-2/β-2 mixed crystals (A, B, C and D) are observed within well-defined areas. At higher concentration, OPP is excluded from (PPP-OPP) _MC_ β^′^-2 and crystallizes separately as (OPP-PPP) _MC_ β-3. Solubility of OPP within (PPP-OPP) _MC_ in β^′^ and β is increased to 45 and 60%, respectively (blue dotted circles).

##### PPP/POO

Considering that PPP remains in α-2 form and that POO is liquid at the tempering temperature (20 °C), the tempered PPP/POO binary system looks very similar to the non-tempered one, excepted that POO crystallizes separately under sub-α-3, α-3 and β^′^-3 forms (C, D and E) (consequence of quenching of the liquid POO). It is mixed with (PPP-POO) _MC_ β-2 (A). A small portion at the left of the phase diagram is showing (PPP-POO) _MC_ β^′^-2 (B). The solubility of POO in PPP (~10%) is somewhat increased after tempering (blue dotted circles).

##### POP/OPP

After the tempering used, both POP and OPP crystallize in L-3: POP transforms from β^′^-2 into β–3 and OPP stabilizes in β^′^-3. As for the non-tempered system, there is still a difference between the left and the right side of the phase diagram, the cut-off also being at ~50/50 ratio, composition of the molecular compound (OPP-POP) _MoC_ observed in dynamic mode. Melting point (~29 °C) of this (OPP-POP) _MoC_ is higher compared to the non-tempered (OPP-POP) _MoC_ (~16 °C), due to β-3 stabilization. One single endotherm is observed at the left side of the molecular compound and two endotherms at the right side. L-3 packings are present within the whole compositional range, mostly corresponding to (POP-OPP) _MC_ β-3 (A) and (OPP-POP) _MC_ β^′^-3 (B).

##### POP/POO

POP stabilizes in β-3, but POO and part of the POO enriched blends were liquid at the tempering temperature (20 °C). This explains the occurrence of POO in sub-α-3 (E), α-3 (D) and β^′^-3(C) mainly at the right part of the system, due to solidification upon quenching. For other compositions, POP/POO ss β^′^-2 clearly transforms into β^′^-3: (POP-POO) _MC_ β^′^-3 (B) is formed. The melting line (~24 °C) (and transition point) corresponds to the transformation into (POP-POO) _MC_ β-3 (A). Solubility of POO into POP as β^′^-3 MC is ~10% (blue dotted lines).

##### OPP/POO

The OPP/POO system is very similar to the POP/POO one and highly simplified compared to direct mode. OPP stabilizes in β^′^-3 at the tempering temperature and part of the blends is partially liquid. The result is the occurrence of (OPP-POO) _MC_ β^′^-3 (A), without transition line as is observed for POP/POO. Upon cooling, the blends crystallize in sub-α-2, α-2 and β^′^-2 as solid solutions (D, C and B), as it is observed for POP/POO. Contrary to POP/POO system, the last melting is (OPP/POO) _MC_ β^′^-3; no β polymorphic form is detected. Solubility of POO into OPP as β^′^-3 MC is ~10% (blue dotted lines).

### 3.4. Ternary Phase Diagrams

Ternary blends were also investigated: ternary diagrams were drawn to supplement the above information. High and low melting iso-lines are reported for the PPP/POP/POO and PPP/OPP/POO systems.

#### 3.4.1. Direct Mode

##### PPP/POP/POO

**Figure 6:**
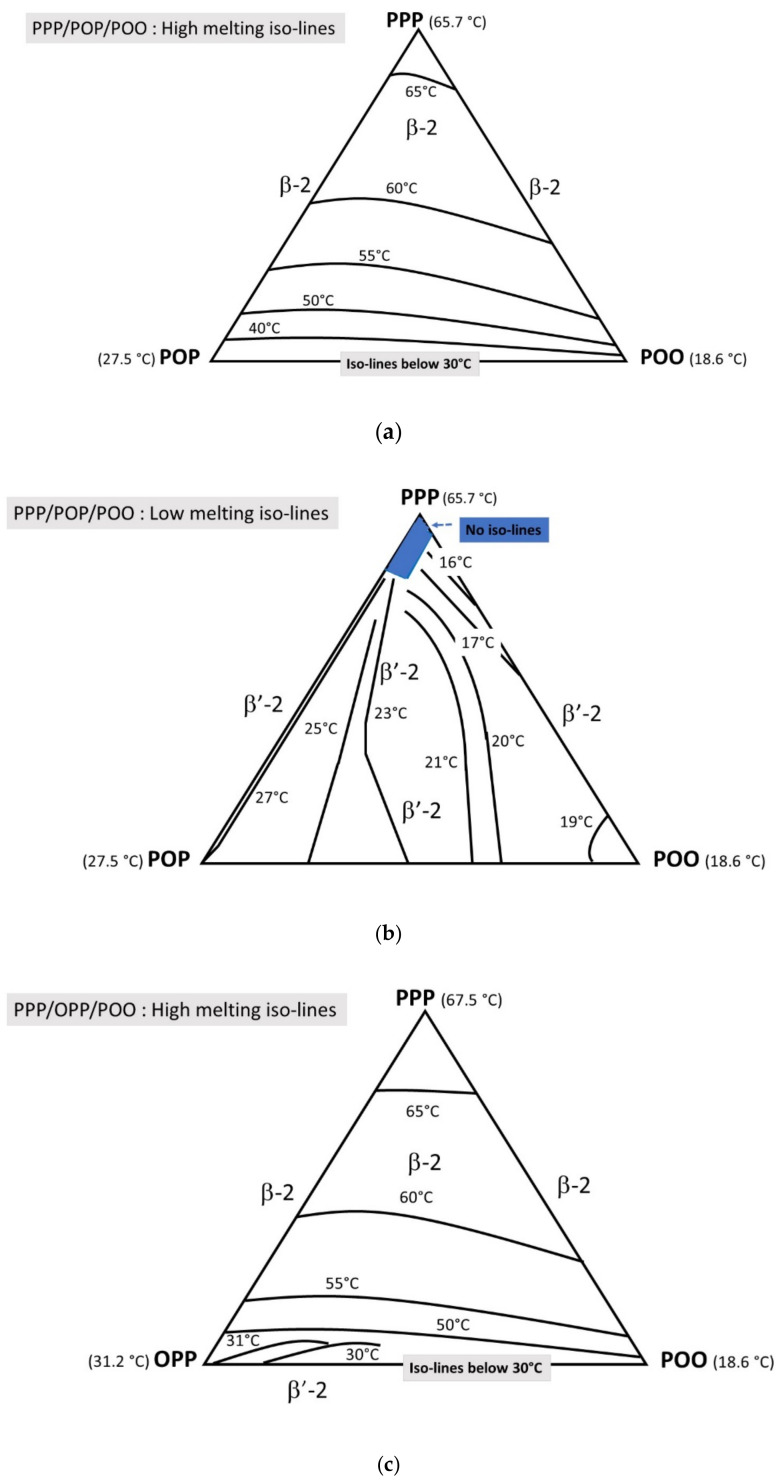

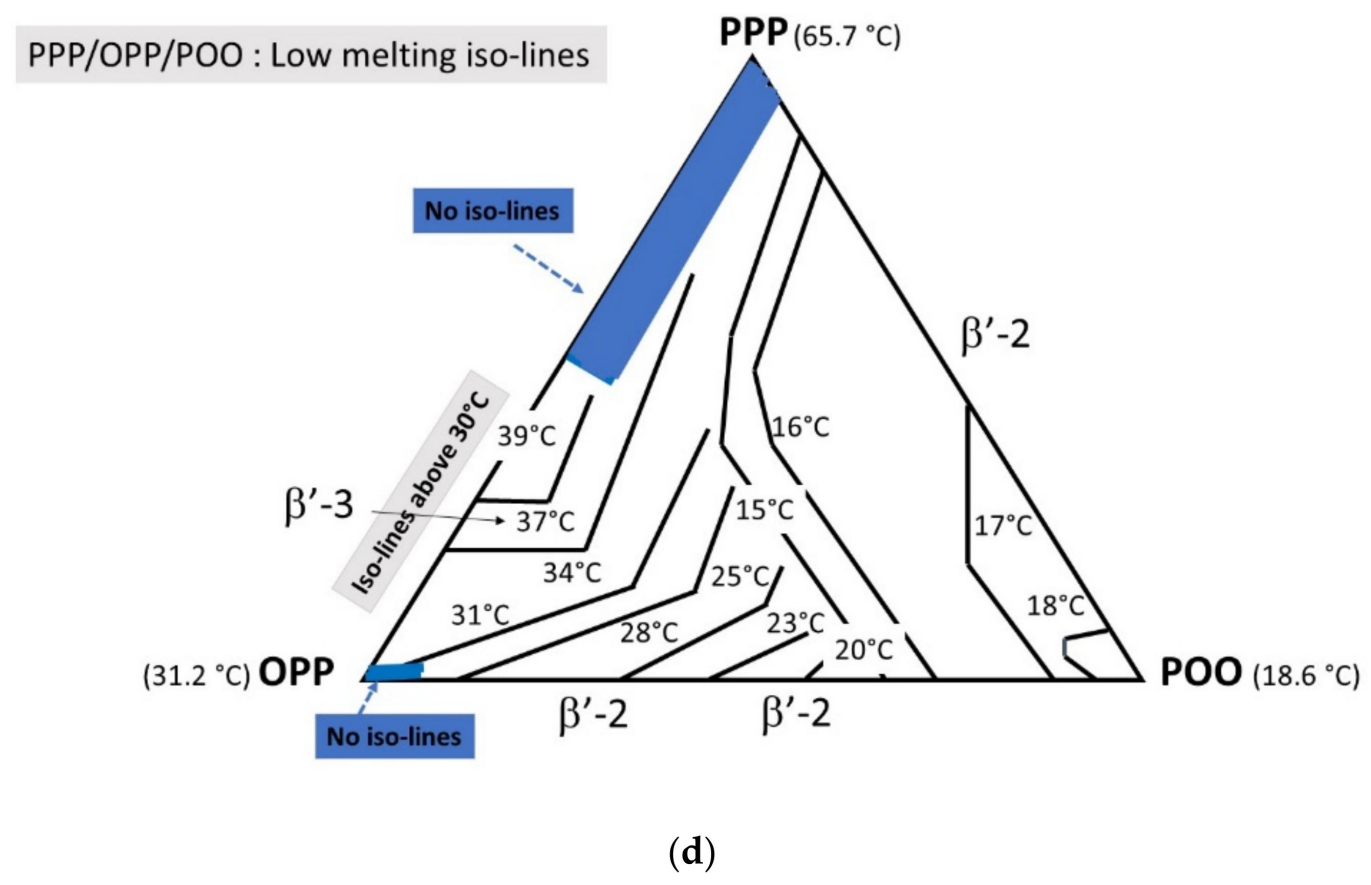
(**a**) (PPP/POP/POO) phase diagram high melting iso-lines; (**b**) (PPP/POP/POO) phase diagram low melting iso-lines; (**c**) (PPP/OPP/POO) phase diagram high melting iso-lines and (**d**) (PPP/OPP/POO) phase diagram low melting iso-lines, in direct mode.

PPP/POP/POO ternary blends exhibit two series of iso-lines: a first series (β melting) between 40 and 65 °C depending on PPP content and a second series (β^′^ melting) between 16 and 27 °C depending on POP and POO relative contents. The high melting iso-lines (Figure 6a) correspond to β-2 polymorphic form; no high-melting iso-lines are detected for POP-POO enriched systems. The low melting iso-lines (Figure 6b) correspond to the monotectic melting in the PPP containing systems or to the last melting in the POP-POO blends; all correspond to β^′^-2 melting. There is a zone, PPP enriched, without low-melting iso-lines due to POP and POO solubility in PPP as mixed crystals.

##### PPP/OPP/POO

PPP/OPP/POO ternary blends also exhibit two series of iso-lines. The high melting iso-lines (Figure 6c) correspond to the ones observed for PPP/POP/POO with β-2 melting temperature increasing from 50 to 65 °C with the amount of PPP. However, due to different co-crystallization properties with POO, some β^′^-2 melting iso-lines (30–31 °C) are observed at OPP/POO enriched compositions. Contrary to PPP/POP/POO, there are clearly two series of low melting iso-lines (Figure 6d). The first series (between 15 and 18 °C) is observed for POO enriched blends (β^′^-2 polymorphism) and the second series (up to 39 °C) corresponds to the melting of OPP enriched blends (β^′^-3 polymorphism).

#### 3.4.2. Tempered Mode

##### PPP/POP/POO

**Figure 7:**
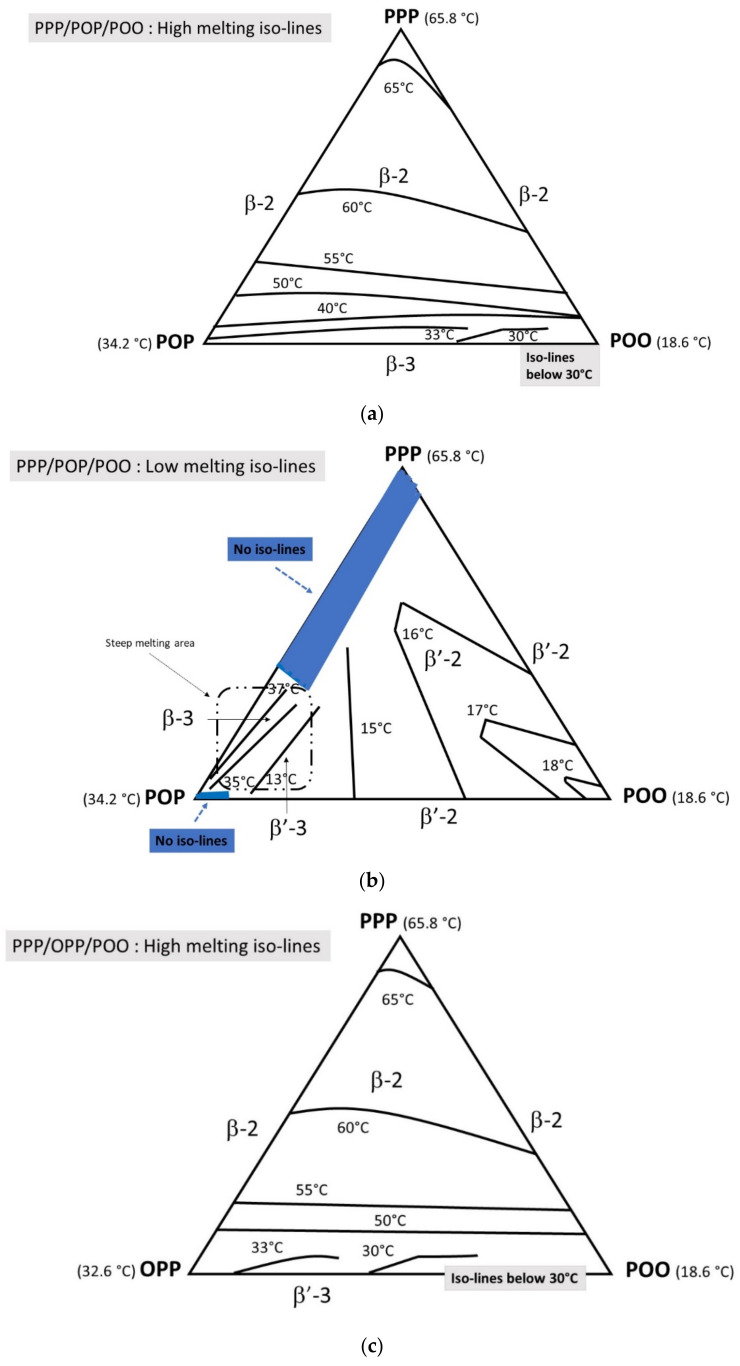

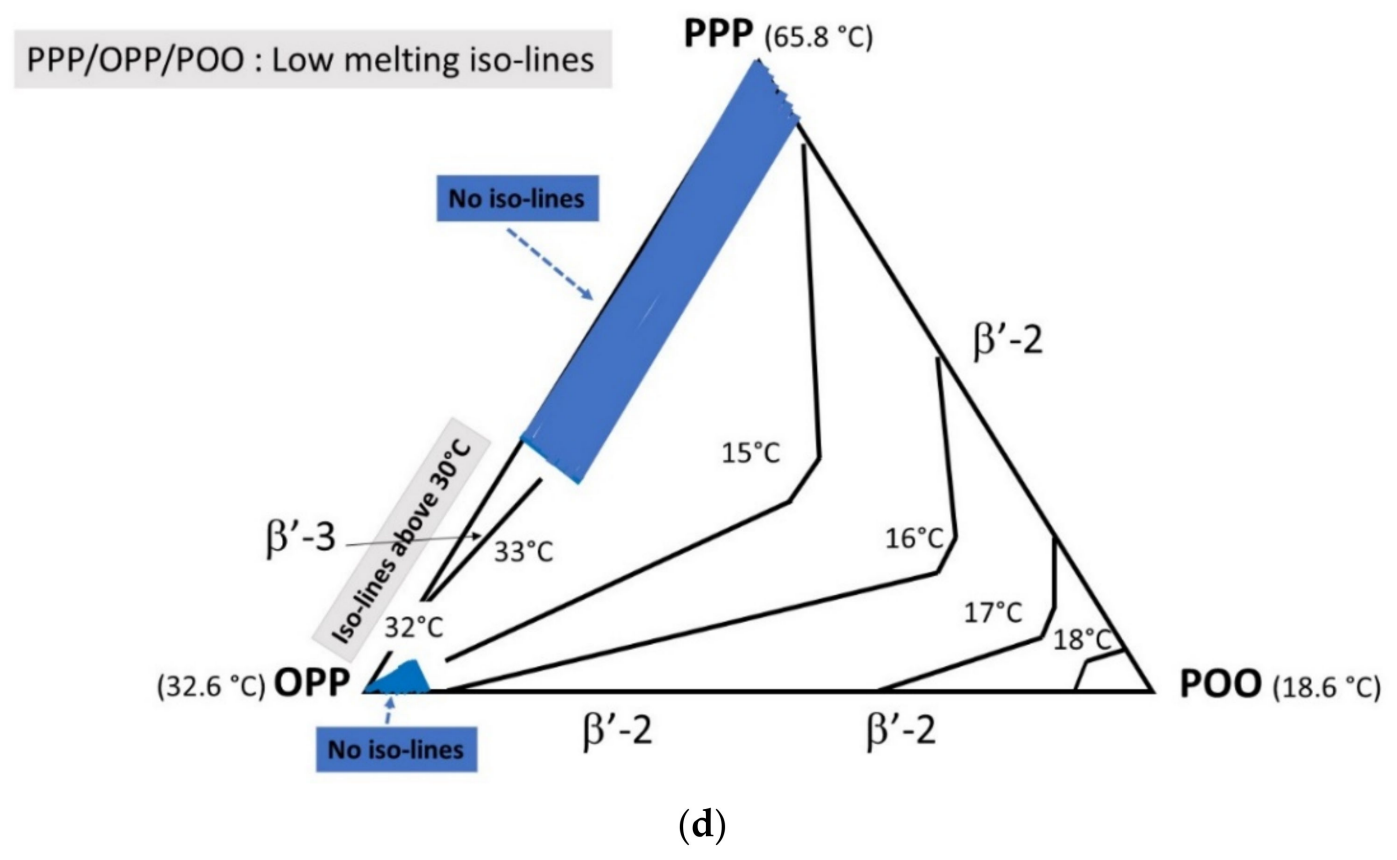
(**a**) (PPP/POP/POO) phase diagram high melting iso-lines; (**b**) (PPP/POP/POO) phase diagram low melting iso-lines; (**c**) (PPP/OPP/POO) phase diagram high melting iso-lines and (**d**) (PPP/OPP/POO) phase diagram low melting iso-lines, in tempered mode.

The high melting iso-lines (Figure 7a) are not highly affected by the used tempering (β-2 melting) except for the POP/POO enriched blends, where supplementary lines appear (30 °C and 33 °C in the diagram) as a consequence of β-3 stabilization. However, the shape of low melting iso-lines (Figure 7b) is substantially modified, mainly in the POP/PPP area; the main reasons are the increase of POP solubility in PPP in β–2 but also the transformation of POP from β^′^2 to the higher melting point β-3 form. Overall, all these modifications create a steep melting area in the enriched POP part of the ternary diagram, which shows β^′^-3 and β-3 polymorphism.

##### PPP/OPP/POO

The high melting iso-lines (Figure 7c) are similar to direct mode (β-2 and β^′^-2 polymorphism). On the contrary, and as for the PPP/POP/POO system, the low melting iso-lines (Figure 7d) are modified. In this case, the low-melting iso-lines remain (between 18 and 15 °C) with only a small portion of OPP enriched blends showing melting lines at about 32–33 °C corresponding to a β^′^-3 polymorphism; this is also a consequence of the increased solubility of OPP in PPP in β^′^-2.

## 4. Discussion

In direct mode, both pure PPP and POP crystallize in double-chain length (L-2) although OPP and POO exhibit a L-3 polymorphism. The binary blends PPP-POP crystallize as L-2 for all the compositions: SS β^′^-2 → MC β^′^-2 → MC β-2. The binary blends PPP-OPP also crystallize as L-2, apart in a restricted portion where the L-3 structure is observed: SS β^′^-2 → MC β^′^-2 + MC β^′^-3 → MC β–2. The binary blends PPP-POO crystallize as L-2 for all the compositions: SS β^′^-2 → MC β-2.

Overall, for the studied systems analyzed in direct mode, PPP can be considered as an inducer of L-2 structures, when it is mixed with POP, OPP or POO; for the last melting form, all binary systems co-crystallize with PPP in L-2 configuration whatever the position or the number of oleic acid on the glycerol. This observation is important since it helps to understand the role of palm oil (as PPP source) to stabilize the crystallization of palm olein in the production of super-olein (step 2) by dry fractionation. In β^′^-2 and β-2 forms, the solubility of OPP in PPP is higher than that of POP and POO. OPP and POP form a β^′^-2 molecular compound at 50/50 concentration ratio, POP and POO exhibit a complete miscibility although OPP and POO also show one molecular compound at 50/50 and one eutectic interaction at 75/25 concentration ratios. OPP tends to induce the formation of molecular compounds when it is blended with POP or with POO.

The tendency of these triacylglycerol blends to form molecular compounds which are stable entities releasing significant heat when crystallizing can explain the typical crystallization behaviors of the olein for the production of super-olein (step 2) and of the soft PMF for the production of hard PMF (step 3) by dry fractionation.

In tempered mode, PPP/POP and PPP/OPP systems show quite similar interactions. The solubility of POP in PPP is significantly increased while that of OPP in PPP does not change much. Polymorphism is L-2, except in the part of the diagrams rich in POP or in OPP. The PPP/POO system is not really modified by the tempering, since at the tempering temperature most of the blends are already in a stable β–2 form or are liquid; solubility of POO in PPP is slightly increased compared to the direct mode. The POP/OPP system evolves into a simpler one, the entire binary system stabilizes in L-3 with a β–3 molecular compound at 50/50 concentration ratio. Last melting of POP/POO and OPP/POO systems also correspond to L-3 structure: β-3 or β^′^-3 as mixed crystals. These phase diagrams are highly similar, the main differences being that the β^′^ → β transition of the mixed crystals is only observed for the POP/POO system; solubility of POO in POP and in OPP is similar. In the PPP containing systems, the changes are not highly pronounced from direct to tempered mode. Larger variations are observed in the systems containing POP, OPP and POO: interactions are simplified in the POP/OPP system, the POP/POO system becomes more complex while the OPP/POO system varies the least.

Regarding the ternary phase diagrams, PPP/POP/POO systems, investigated in direct mode, behave quite similarly to the PPP/OPP/POO ones, in what concerns the highest melting iso-lines. Polymorphism is L-2 all over the phase diagrams. However, POP-POO and OPP-POO blends present different melting behaviors, creating huge differences in the low-melting iso-lines. Although PPP/POP/POO low melting iso-lines all correspond to L-2 polymorphism, low-melting iso-lines of part of the PPP/OPP/POO blends correspond to L-3 polymorphism. In tempered mode, both PPP/POP/POO and PPP/OPP/POO ternary diagrams present an area showing L-3 polymorphism for the high melting iso-lines: β-3 for the POP system and β^′^-3 for the OPP. An L-3 area is also observed in both systems for the low melting iso-lines: β-3 and β^′^-3 for PPP/POP/POO and β-3 for PPP/OPP/POO.

## 5. Summary

The results show that the unsaturated triacylglycerols are poorly soluble in the fully saturated one (PPP), that intersolubility decreases in proportion to the number of unsaturated fatty acids, that positional isomerism (POP/OPP) has a major impact, that OPP may induce formation of molecular compounds and that co-crystallization properties are highly modified by tempering depending on the polymorphic properties of the systems. The results also explain some crystallization behaviors observed during the dry fractionation of palm oil. Adding palm oil as PPP source is useful to ensure a reproducible olein crystallization since PPP induces stable L-2 structures in systems containing P_2_O and PO_2_. The difficulty to control the heat release observed during olein crystallization and during soft PMF crystallization has been shown to be linked to the formation of molecular compounds at certain concentration ratio in systems containing POP and OPP. Any solution (modified refining, specific blending, ...) allowing to modify this ratio in palm oil or fractions are in favor of better control of the molecular interactions involved.

## Figures and Tables

**Table 1:** PPP, P_2_O and PO_2_ contents (% by HPLC) obtained in some fractions from multi-step dry fractionation of palm oil. N.D.: not detectable; IV: iodine value.

Triacylglycerol (%)	Palm Oil IV 51–53	Olein IV 56–57	Super-Olein IV 64–65	Top-Olein IV 71–73	Stearin IV 30–32	Super-Stearin IV 9–14	Soft PMF IV 44–46	Hard PMF IV 32–34
PPP	4–6	N.D.	N.D.	N.D.	25–27	65–70	<1	<2
P_2_O	27–29	28–30	17–18	7–9	27–29	8–12	47–48	65–67
PO_2_	20–22	22–24	29–30	34–36	10–11	2–4	12–13	2–3

**Table 2:** Melting (endotherms) and transition (exotherms) temperatures and polymorphic forms in direct mode; last melting temperatures (endotherms) and polymorphic forms in tempered mode for pure PPP, POP, OPP and POO.

	Direct Mode				Tempered Mode
PPP	44.9 °C	47.4 °C	52.4 °C	65.7 °C	65.8 °C
	endotherm	exotherm	exotherm	endotherm	endotherm
	α-2 form		β^′^-2 form	β-2 form	β-2 form
POP	14.1 °C		27.5 °C		34.2 °C
	exotherm		endotherm		endotherm
	α-2 form		β^′^-2 form		β-3 form
OPP	-	19.0 °C	23.3 °C	31.2 °C	32.6 °C
	-	endotherm	exotherm	endotherm	endotherm
	sub-α-3 form	α-3 form		β-3 form	β^′^-3form
POO	−14.3 °C		3.1 °C	18.6 °C	Same as in direct mode
	exotherm		exotherm	endotherm	
	sub-α-3 form		α-3 form	β^′^-3 form	

**Table 3:** Short and long spacings observed for POP, OPP and POO in direct mode (m: medium intensity; S: strong intensity; VS: very strong intensity).

**POP**	**α-2**	**sub–β^′^-2**	**β^′^-2**
Short spacings (Å)	4.2 (VS)	4.4 (S) 4.2 (S) 4.9 (m)	5.0 (m) 4.5 (m) 4.4 (S) 4.2 (m) 4.0 (VS)
Long spacings (Å)	50.1	45.1	43.2
**OPP**	**sub-α-3**	**α-3**	**β^′^-3**
Short spacings (Å)	4.2 (S) 3.8 (m)	4.2 (VS)	4.7 (m) 4.4 (m) 4.1 (VS) 3.8 (S)
Long spacings (Å)	82.9	81.1	71.3
**POO**	**sub-α-3**	**α-3**	**β^′^-3**
Short spacings (Å)	4.3 (S) 3.9 (m)	4.2 (VS)	4.6 (m) 4.4 (m) 3.9 (S)
Long spacings (Å)	68.3	67.0	66.5
